# Supplementary material for: Evaluating metabolic and genomic data for predicting grain traits under high night temperature stress in rice
Source: G3 (Bethesda). 2023 Mar 7;13(5):jkad052. doi: 10.1093/g3journal/jkad052 (PMC10151405; doi:10.1093/g3journal/jkad052)
Supplement: jkad052_Supplementary_Data [file jkad052_supplementary_data.pdf]

Evaluating metabolic and genomic data for predicting grain traits under high night temperature stress in rice

Ye Bi[1], Rafael Massahiro Yassue[1,2], Puneet Paul[3], Balpreet Kaur Dhatt[3], Jaspreet Sandhu[3], Thi Phuc Do[4,5], Harkamal Walia[3], Toshihiro Obata[5], and Gota Morota[*,1,6]

[1]School of Animal Sciences, Virginia Polytechnic Institute and State University, Blacksburg, VA, 24061, USA

[2]Department of Genetics, 'Luiz de Queiroz' College of Agriculture, University of São Paulo, São Paulo, 13418, Brazil

[3]Department of Agronomy and Horticulture, University of Nebraska-Lincoln, Lincoln, NE, 68583, USA

[4]Faculty of Biology, VNU University of Science, Vietnam National University, 334 Nguyen Trai, Thanh Xuan, Hanoi, Vietnam

[5]Department of Biochemistry, University of Nebraska-Lincoln, Lincoln, NE, 68588, USA

[6]Center for Advanced Innovation in Agriculture, Virginia Polytechnic Institute and State University, Blacksburg, VA, 24061, USA

Tables

Table S1: List of metabolites.

Number	Metabolite	Number	Metabolite
1	hexanoic acid	2	alanine
3	valine	4	urea
5	ethanolamine	6	leucine
7	glycerol	8	nicotinic acid
9	isoleucine	10	proline
11	glycine	12	glyceric acid
13	citraconic acid	14	serine
15	threonine	16	Beta alanine
17	malic acid	18	dihydrouracil
19	threitol	20	methionine
21	aspartic acid	22	cytosine
23	trans-4-hydroxy proline	24	gamma-aminobutyric acid (GABA)
25	glutamic acid	26	hydroxybenzoic acid
27	asparagine	28	arabinose
29	lyxose	30	ribose
31	xylitol	32	arabitol
33	ribitol	34	diglycerol
35	4-hydroxy-3-methoxybenzoic acid	36	glycerol-1 phosphate
37	glutamine	38	dihydroxybenzoic acid
39	ornithine	40	citrulline
41	citric acid	42	adenine
43	fructose-1	44	allantoin-2
45	altrose	46	lysine
47	histidine	48	glucose
49	tyrosine	50	mannitol
51	sorbitol	52	indoleacetic acid
53	pantothenic acid	54	glucosaminic acid
55	allantoin-3	56	ferulic acid
57	N-acetyl-D-glucosamine	58	allo-inositol
59	tryptophan	60	3.5-dimethoxy-4-hydroxycinnamic acid
61	uridine	62	eicosapentaenoic acid
63	adenosine	64	trehalose
65	maltose	66	sophorose
67	catechin	68	melibiose
69	isomaltose	70	galactinol
71	phosphoric acid	72	Sucrose
73	Raffinose		

Table S2: Conventional heritability estimates of grain size phenotypes in control and high night temperature (HNT) stress conditions.

Treatment	Grain length	Grain width	Perimeter
Control	0.71	0.64	0.50
HNT	0.74	0.73	0.54

Table S3: Predictive correlations of grain-size phenotypes using metabolic, genomic, and multi-omic predictions in control and high night time temperature stress conditions.

Source	Model	Grain length		Grain width		Grain perimeter	
		Control	HNT[8]	Control	HNT	Control	HNT
Metabolites	MOLS[1]	0.20	0.14	0.26	0.36	0.14	0.10
	MBLUP[2]	0.33	0.31	0.54	0.54	0.32	0.27
	BayesC	0.35	0.33	0.52	0.54	0.35	0.29
	RF[3]	0.25	0.29	0.57	0.54	0.21	0.26
	SVR[4]	0.29	0.29	0.52	0.50	0.28	0.24
	XGBoost[5]	0.27	0.29	0.53	0.47	0.28	0.29
Genetic markers	GBLUP[6]	0.64	0.64	0.73	0.67	0.57	0.63
Genetic markers & Metabolites	GMBLUP[7]	0.64	0.63	0.73	0.70	0.58	0.61

[1] Metabolic ordinary least squares
[2] Metabolic best linear unbiased prediction
[3] Random forest
[4] Support vector regression
[5] Extreme gradient boosting
[6] Genomic best linear unbiased prediction
[7] Genomic metabolic best linear unbiased prediction
[8] High night time temperature

Table S4: Predictive correlations of leave-one-subpopulation-group-out cross-validation in control and high night temperature stress conditions.

Testing set[1]	Grain length			Grain width			Grain perimeter		
	MBLUP[2]	GBLUP[3]	GMBLUP[4]	MBLUP	GBLUP	GMBLUP	MBLUP	GBLUP	GMBLUP
Control									
tropical-japonica	0.03	0.23	0.22	0.16	0.18	0.29	0.28	0.08	0.54
temperate-japonica	-0.18	0.69	0.38	0.24	0.00	0.10	-0.20	0.62	0.34
indica	0.38	0.68	0.72	-0.09	0.17	0.07	0.36	0.51	0.50
aus	0.21	0.46	0.45	0.29	0.28	0.29	0.32	0.32	0.51
admixed-japonica	0.09	0.31	0.26	0.12	0.39	0.41	0.15	0.04	0.17
High night temperature									
tropical-japonica	0.18	0.31	0.33	0.53	0.43	0.64	0.28	0.27	0.37
temperate-japonica	0.06	0.66	0.53	0.40	0.01	0.03	0.08	0.63	0.46
indica	0.59	0.36	0.40	0.10	0.37	0.14	0.51	0.25	0.45
aus	0.05	0.30	0.29	0.09	-0.26	0.05	0.07	0.33	0.34
admixed-japonica	0.23	0.50	0.57	0.02	0.52	0.57	0.09	0.49	0.45

[1] Aromatic, admixed indica, and admixed were not used as the testing set because the number of genotypes was less than 10

[2] Metabolic best linear unbiased prediction

[3] Genomic best linear unbiased prediction

[4] Genomic metabolic best linear unbiased prediction

Figures

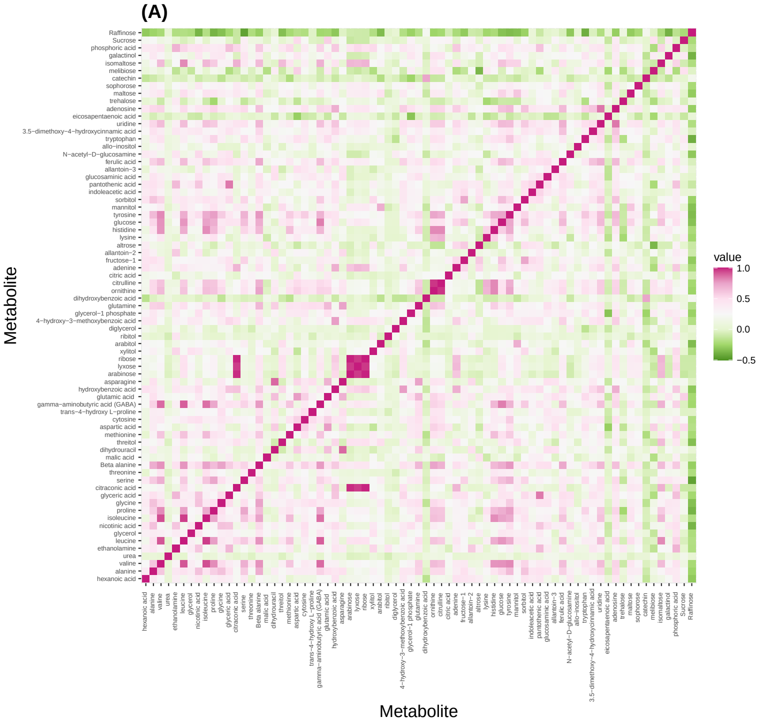

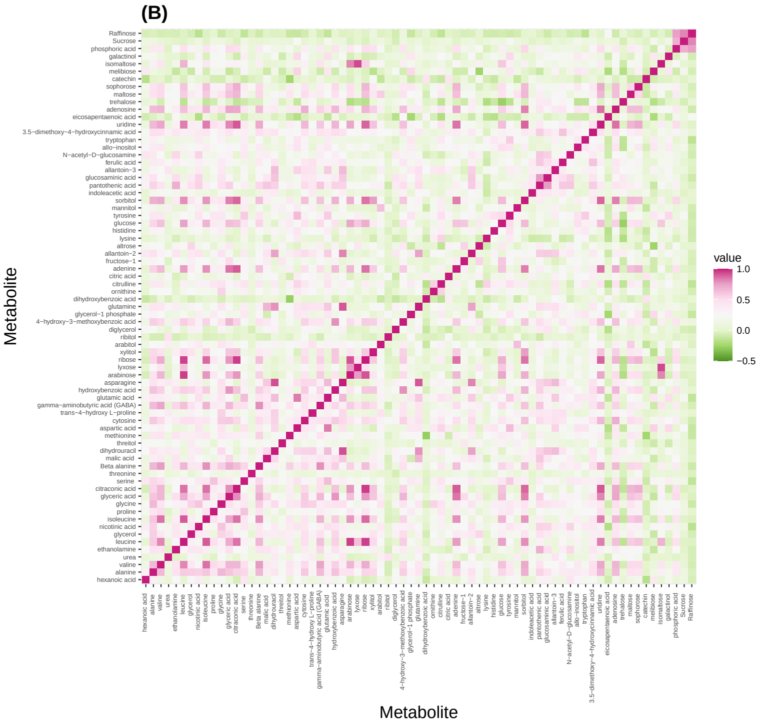

Figure S1: Pearson correlation heat map among metabolic profiles in control (A) and high night time temperature stress conditions (B).

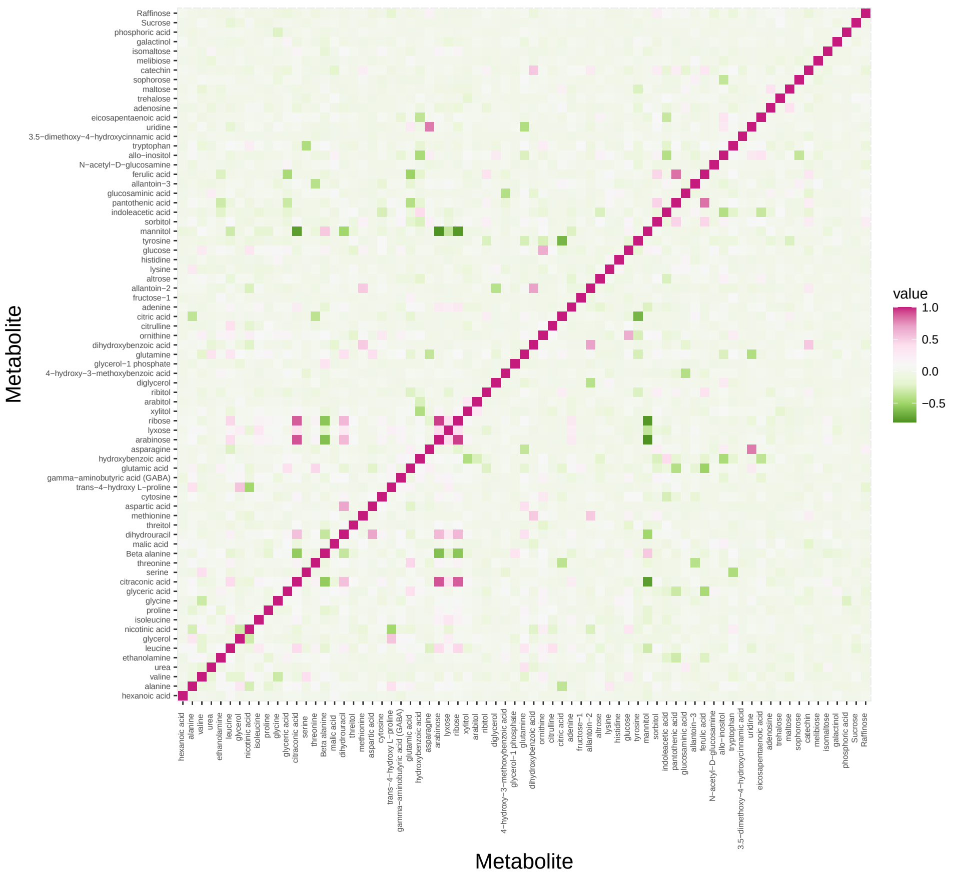

Figure S2: Pearson correlation heat map among metabolic profiles expressed as the ratio of control and high night time temperature stress.

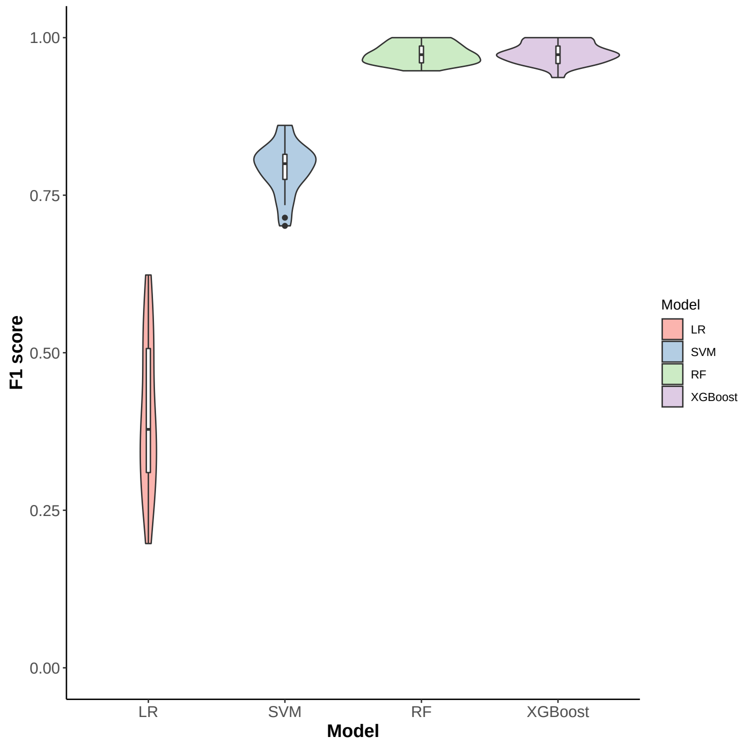

Figure S3: F1 scores of high night time temperature conditions (control and stress) using 73 metabolites. LR: logistic regression; SVM: support vector machine; and RF: random forest; XGBoost: extreme gradient boosting.

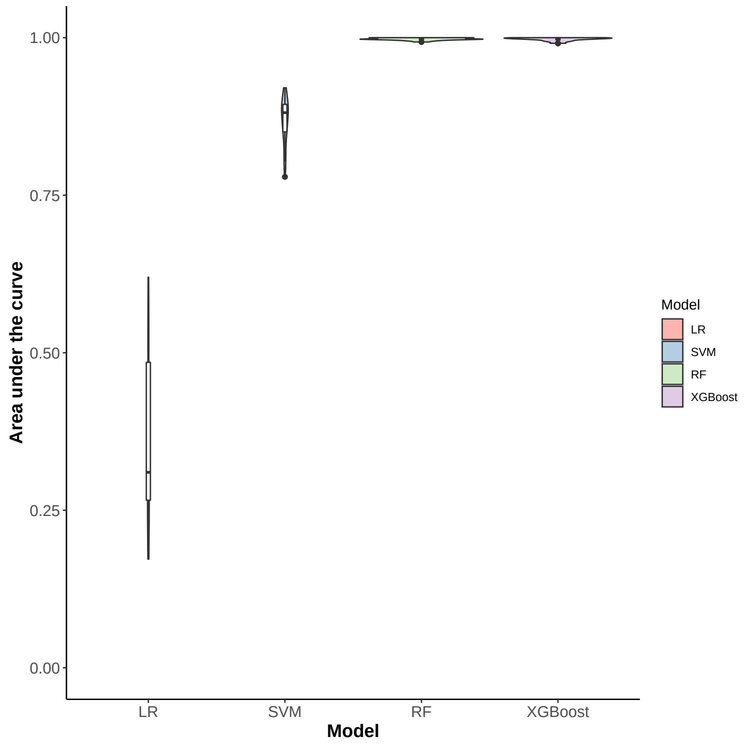

Figure S4: Area under the curve of high night time temperature conditions (control and stress) using 73 metabolites. LR: logistic regression; SVM: support vector machine; and RF: random forest; XGBoost: extreme gradient boosting.

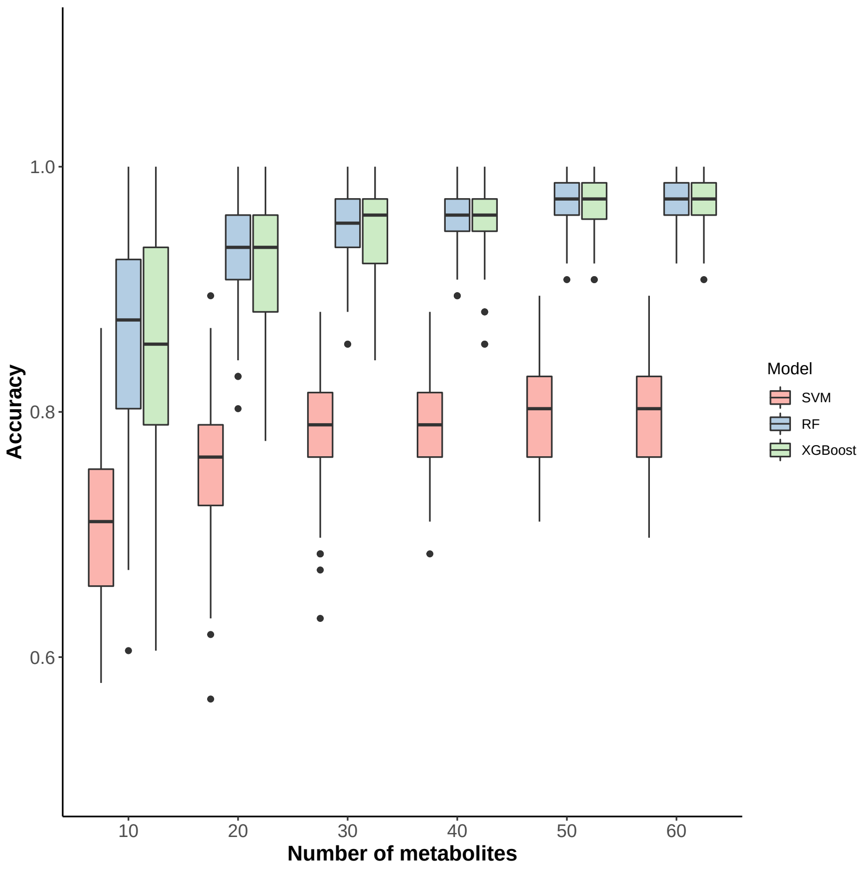

Figure S5: Classification accuracy of high night time temperature conditions (control and stress) using different number of metabolites using support vector machine (SVM), random forests (RF), and extreme gradient boosting (XGBoost).

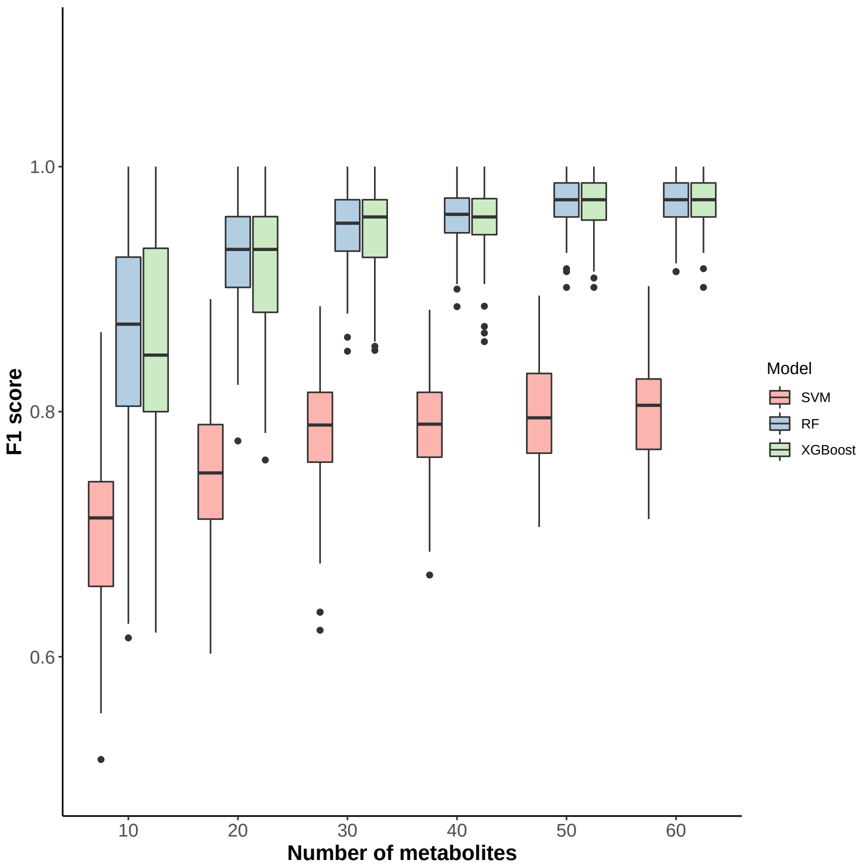

Figure S6: F1 scores of high night time temperature conditions (control and stress) using different number of metabolites using support vector machine (SVM), random forests (RF), and extreme gradient boosting (XGBoost).

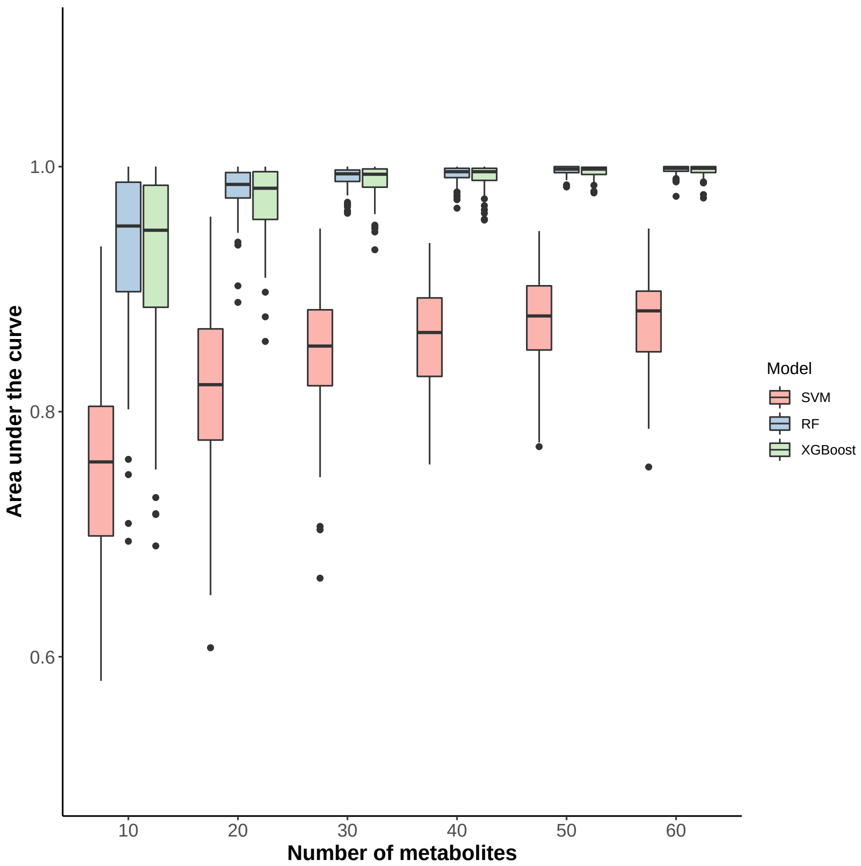

Figure S7: Area under the curve of high night time temperature conditions (control and stress) using different number of metabolites using support vector machine (SVM), random forests (RF), and extreme gradient boosting (XGBoost).

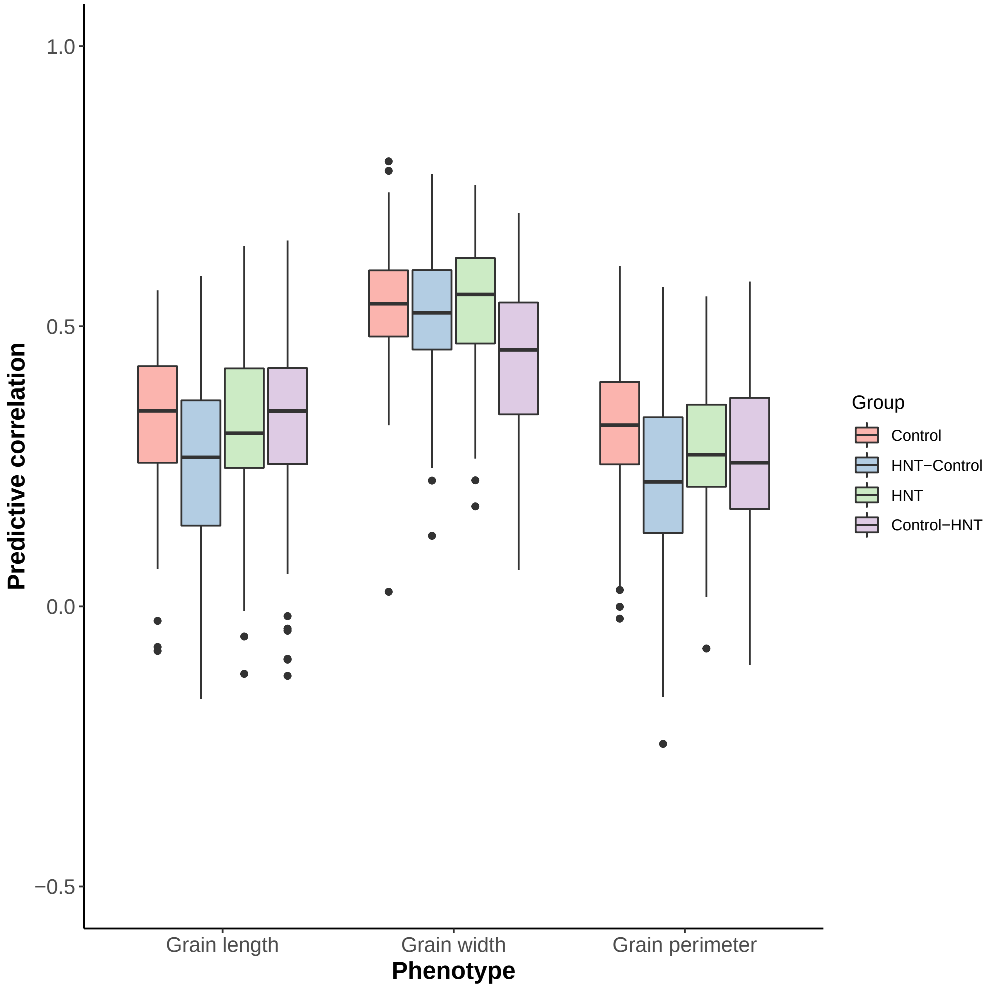

Figure S8: Metabolic predictions across high night time temperature (HNT) stress conditions. Control: Train the model using phenotypes and metabolites in control and predict control phenotypes from metabolites in control conditions. Control-HNT: Train the model using phenotypes and metabolites in control and predict HNT phenotypes from metabolites in HNT conditions using the metabolic effect estimated in control conditions. HNT: Train the model using phenotypes and metabolites in HNT and predict HNT phenotypes from metabolites in HNT conditions. HNT-Control: Train the model using phenotypes and metabolites in HNT and predict phenotypes from metabolites in control conditions using the metabolic effect estimated in HNT condition.

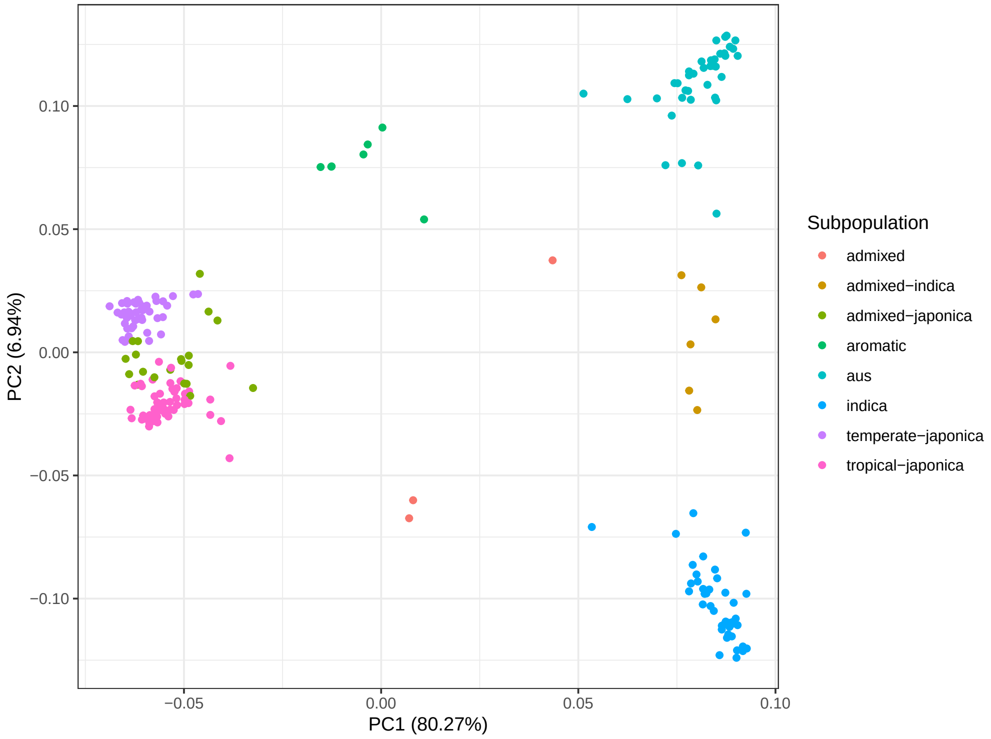

Figure S9: Principal component analysis plot based on the genomic relationship matrix.

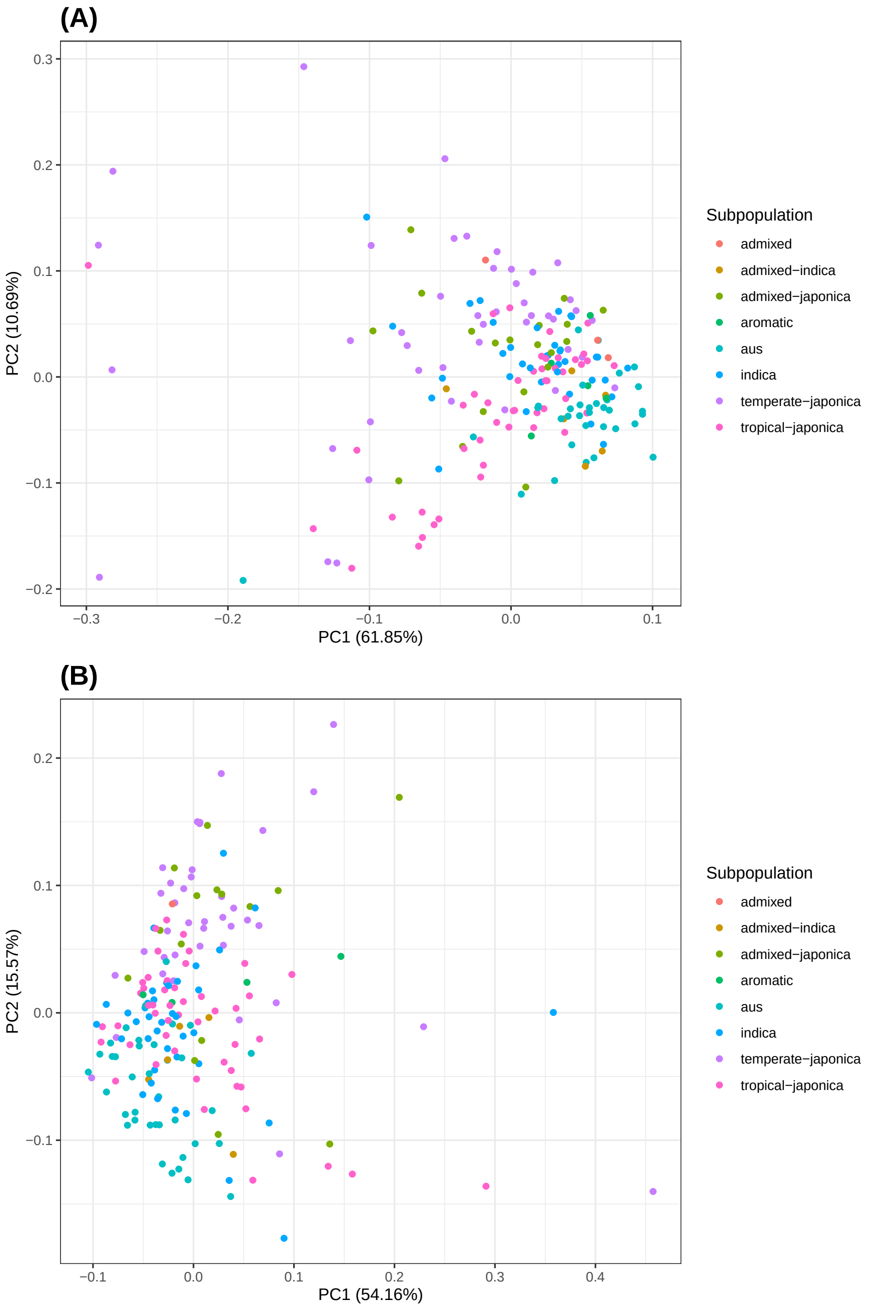

Figure S10: Principal component analysis plot based on the metabolic relationship matrix in control (A) and high night time temperature stress conditions (B).
